# YASARA View—molecular graphics for all devices—from smartphones to workstations

**DOI:** 10.1093/bioinformatics/btu426

**Published:** 2014-07-04

**Authors:** Elmar Krieger, Gert Vriend

**Affiliations:** Centre for Molecular and Biomolecular Informatics, NCMLS, Radboud University Nijmegen Medical Centre, 6500 HB Nijmegen, the Netherlands

## Abstract

**Summary:** Today's graphics processing units (GPUs) compose the scene from individual triangles. As about 320 triangles are needed to approximate a single sphere—an atom—in a convincing way, visualizing larger proteins with atomic details requires tens of millions of triangles, far too many for smooth interactive frame rates. We describe a new approach to solve this ‘molecular graphics problem’, which shares the work between GPU and multiple CPU cores, generates high-quality results with perfectly round spheres, shadows and ambient lighting and requires only OpenGL 1.0 functionality, without any pixel shader *Z*-buffer access (a feature which is missing in most mobile devices).

**Availability and implementation:** YASARA View, a molecular modeling program built around the visualization algorithm described here, is freely available (including commercial use) for Linux, MacOS, Windows and Android (Intel) from www.YASARA.org.

**Contact:** elmar@yasara.org

**Supplementary information:** Supplementary data are available at *Bioinformatics* online.

## 1 INTRODUCTION

In 1966, Cyrus Levinthal pioneered molecular graphics at the Massachusetts Institute of Technology, when he set up the first interactive wire-frame display of a protein on a monochrome oscilloscope (Levinthal, 1966). Since then, molecular graphics has made tremendous progress, mostly thanks to the video game industry, which induced the rise of graphics processing units (GPUs). Today many different molecular visualizers are available, e.g. VMD (Humphrey *et al.*, 1996), Chimera (Pettersen *et al.*, 2004), PyMol (DeLano, 2005) or QuteMol (Tarini *et al.*, 2006), each using different tricks to boost rendering performance and quality. We describe an algorithm that can cope with two specific difficulties: first, it does not depend on high-end shader tricks and thus works on smartphones too. And second, it does not require expensive precalculation steps that depend on atom positions. It can thus visualize moving atoms, allowing to perform interactive molecular dynamics simulations on smartphones and tablets.

## 2 METHODS

**Fig. 1.**
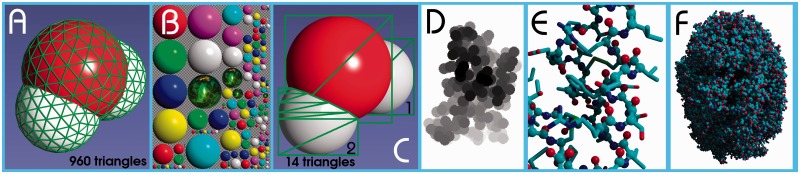
A water molecule rendered classically with 960 triangles (**A**) or quickly using texture mapping and precalculated impostors arranged in a single texture (**B**). The texture contains ray-traced images of spheres with various colors (two of which are blended with a variable factor to create other colors and color gradients) and various sizes (so-called ‘mipmaps’, which reduce aliasing artifacts). The spheres coated with a stellar nebula are used to draw atoms selected by the user. The gray checkerboard indicates transparent pixels. Using texture (B), the water molecule in (A) can be drawn quickly using just 14 triangles (**C**). Low-resolution depth map of PDB file 1CRN to calculate shadows (**D**), balls and sticks of 1CRN (**E**) and space-filling display of PDB file 1AON (**F**)

The general idea is very simple and has been used ever since texture mapping became part of 3D graphics: if an object is too complex (like the 960 triangles required to draw a single water molecule in Fig. 1A) it is replaced with ‘impostors’, i.e. fewer triangles that have precalculated textures attached, which make them look like the original object. Texture mapping means that a triangle is not rendered with a single color, but an image (the texture) is attached to it instead. For each of the three triangle vertices, the programmer can specify the corresponding 2D coordinates in the texture, and the hardware interpolates in between. So, instead of drawing 320 triangles to create one, still somewhat edgy atom, we simply draw the precalculated image of a perfectly round atom. As textures may be partly transparent, this image can be drawn as a simple square (transparent in the corners), which requires just two triangles.

In practice, many different images of atoms are needed, as atoms can have different colors and sizes. Regarding colors, we use blue, magenta, red, yellow, green, cyan and gray, and blend any two of them with a variable blending factor to support color gradients. Regarding sizes, the precalculated images can be shrunk on the fly during texture mapping, but the shrinking procedure reduces the image quality. That is why multiple smaller images of each atom are stored as well. Changing the texture during rendering reduces performance, and consequently all these atom images are squeezed into a single texture of size 1024 × 1024, which is shown in Figure 1B. When the user changes the position of the light source, this texture is updated from a collection of 200 different views, prerendered with www.POVRay.org. For stereo graphics, a second texture is used that has the atoms prerendered from a slightly shifted point of view.

This straightforward approach has not been routinely used in the past for the following reason: when the GPU draws a pixel, it stores its *Z*-value (the distance from the view-plane) in the *Z*-buffer, and then never again draws a pixel at this location, unless it has a smaller *Z*-value. When spheres are modeled with lots of triangles as in Figure 1A, each pixel has the right *Z*-value associated, so that the spheres intersect correctly thanks to the *Z*-buffer. With our shortcut, however, each sphere consists of just two triangles that are parallel to the view-plane, and each pixel of the sphere image thus has the same *Z*-value. Consequently, the spheres do not intersect at all, instead the closer one completely occludes the more distant one. This is fine when drawing non-intersecting spheres (in sticks and balls and sticks visualization styles), but obviously goes wrong with a space-filling style. The logical solution would be to adjust the pixel *Z*-values on the fly during rendering (with a so-called ‘pixel shader’), but this approach is either slow (because the hardware can no longer perform an early *Z*-test to discard pixels) or not supported at all (e.g. mobile devices based on OpenGL ES lack this feature, and PowerVR GPUs do not even have a *Z*-buffer). The algorithm described here therefore takes a different route; it shares the work between central processing unit (CPU) and GPU according to the following recipe, which can easily be distributed over multiple CPU cores (a very detailed 20-page step-by-step recipe has been included as Supplementary Material):

1. The CPU transforms the atom coordinates from object space to screen space and immediately discards atoms that are offscreen.
2. For each atom i, the CPU creates a temporary *Z*-buffer that includes atom i and all the more distant atoms k, which can influence the shape of atom i by intersection, i.e. those atoms whose sphere image touches atom i and who are closer along *Z* than their own radius R_k_. The atoms k could be found quickly with a neighbor search grid, but it turns out that the trivial approach to just look at covalently bound atoms is good enough.
3. Finally, the CPU loops over the pixel lines in the temporary *Z*-buffer of atom i, checks which lines are affected by intersections and emits a number of triangles that trace these intersections. The principle is clarified in Figure 1C, which shows how to draw a water molecule with just 14 triangles instead of 960 triangles.
4. If atoms are shown as sticks or balls and sticks, cylinders need to be drawn that connect the atoms (Fig. 1E). To reduce the polygon count, only the front side of the cylinders is drawn, using between 2 and 18 triangles, depending on the distance from the viewer. Cylinders always use the same texture as the atoms (Fig. 1B), which ensures visual continuity.
5. Shadows and ambient lighting are calculated per atom, not per pixel. The CPU first draws a low-resolution depth map of the scene where atoms have a diameter of just 15 pixels (Fig. 1D), either seen from the position of the light source (shadows) or from the six main directions (ambient lighting). Then it integrates the amount of light reaching each atom (i.e. the fraction of pixels not occluded by closer ones) and darkens the atom accordingly (using either GL_EXT_fog_coord or multi-texturing).

This fast way of drawing molecules also has three limitations compared with the classic approach: First, atom colors must be mixed from two of the standard colors present in the texture (Fig. 1B), which allows to create most useful colors, but not all colors. Second, the maximum atom size on screen is limited to the largest atom size in the texture (currently, 256 × 256 pixels), unless one wants to use lower quality upscaled atoms. To prevent atoms from getting too small, YASARA therefore restricts its window size to Full HD, but we plan to double the texture size to 2048 × 2048 soon, covering 4 K and similar hires displays. And third, drawing transparent atoms is not straightforward and currently not implemented.

## 3 RESULTS

A visualization example for the chaperonin GroEL/ES (1AON, 58 884 atoms) with real-time shadows is shown in Figure 1F. On a 240 EUR Motorola Razr i smartphone (Intel Atom Z2480@2 GHz with two threads and PowerVR SGX 540, 960 × 540 pixels, Android 4) the algorithm reaches 4–12 frames per second, depending on the number of atoms on screen (or 5–30 fps with ambient lighting but no shadows). This is about 10× as fast as other popular apps (which, however, do not support shadows). On a Windows 8 tablet with the faster Atom Z2760 CPU@1.8 GHz, four threads, 1366 × 768 pixels and PowerVR SGX 545, the frame rate ranges from 8–15 fps (12–30 fps without shadows, about 6× as fast as others). On a high-end workstation, the frame rate is usually above the refresh rate of the screen (60 Hz) for all but the largest structures (ribosomes etc.). We separately tested the usability for interactive molecular dynamics (not in YASARA View) and obtained 4 fps on the Motorola Razr i for Dihydrofolate reductase in water (23 788 atoms), 7.9 A VdW cutoff and PME electrostatics, just enough to pull atoms around.

## Supplementary Material

Supplementary Data
