# Supplementary material for: YASARA View—molecular graphics for all devices—from smartphones to workstations
Source: Bioinformatics. 2014 Jul 4;30(20):2981–2. doi: 10.1093/bioinformatics/btu426 (PMC4184264; doi:10.1093/bioinformatics/btu426)
Supplement: Supplementary Data [file supp_30_20_2981__index.html]

YASARA View – molecular graphics for all devices – from smartphones to workstations — YASARA View—molecular graphics for all devices—from smartphones to workstations — YASARA View—molecular graphics for all devices—from smartphones to workstations — Supplementary Data 

# YASARA View—molecular graphics for all devices—from smartphones to workstations

## Supplementary Data

files

**Files in this Data Supplement:**

- Supplementary Data - pdf file
