# Supplementary material for: YASARA View—molecular graphics for all devices—from smartphones to workstations
Source: Bioinformatics. 2014 Jul 4;30(20):2981–2. doi: 10.1093/bioinformatics/btu426 (PMC4184264; doi:10.1093/bioinformatics/btu426)
Supplement: Supplementary Data [file supp_btu426_YASARAViewAlgorithm.pdf]

YASARA View - molecular graphics for all devices - from smartphones to workstations

by Elmar Krieger and Gert Vriend

This accessory document for our Bioinformatics Applications Note contains the detailed step-by-step recipe to implement the visualization algorithm described. This includes the creation of ray-traced spheres with POVRay, the setup steps and the actual application main cycle.

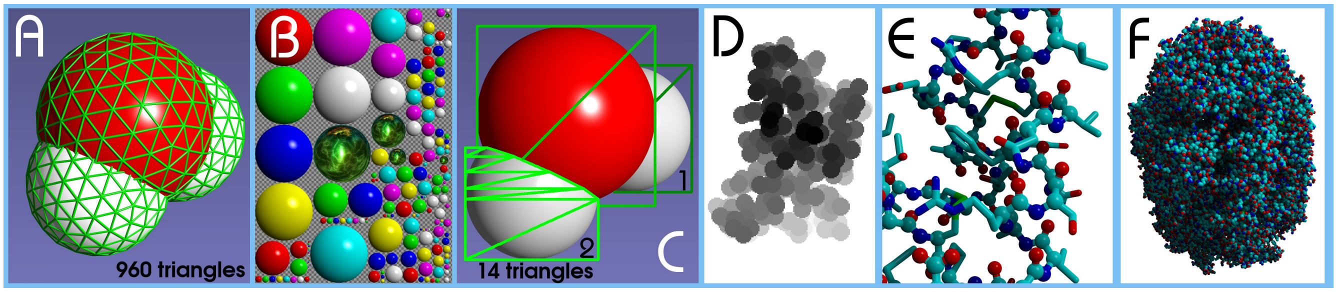

1 Prepare the impostor graphics (=sphere images), for example using POVRay:

1.1 Loop over the S different sphere styles. We use eight styles, each described by a different POVRay texture: Normal with phong shading, normal without phong shading, sky reflection with phong shading, normal with phong shading and bumps, agate, crackle, granite and outline (the latter means single color atoms with a black border to yield comic-style graphics, as in the PDB's molecule of the month):

```
char *pov_texture[]={
  /* 0 Normal with phong shading */
  "texture { pigment { color Ball } finish { phong 1 ambient 0.15 } }",
  /* 1 Normal without phong shading */
  "texture { pigment { color Ball } finish { ambient 0.15 } }",
  /* 2 Sky reflection with phong shading */
  "texture { pigment { granite turbulence 0.70 color_map { [.4, color Ball ] [1.0,
   color   1.3*Ball ] } scale 0.8 } finish { phong 1 ambient 0.15 } } ",
  /* 3 Normal with phong shading and bumps */
  "texture { pigment { color Ball } normal { bumps 0.25 scale 0.05 } finish { phong 1
   ambient 0.15 } }",
```

```
/* 4 Agate (see http://astronomy.swin.edu.au/~pbourke/povray/bump/) */
"texture { pigment { color Ball } normal { agate agate_turb 0.5 scale 1 } finish {
 phong 1 ambient 0.15 } }",
/* 5 Crackle */
"texture { pigment { color Ball } normal { crackle 1 scale 0.2 } finish { phong 1
 ambient 0.15 } }",
/* 6 Granite */
"texture { pigment { color Ball } normal { granite scale 0.5 } finish { phong 1
 ambient 0.15 } }",
/* 7 Outline (dummy, needs post-processing) */
"texture { pigment { color Ball } finish { ambient 1.0 } }" };
```

1.1.1 Open a binary file to store the sphere bitmaps with the current style (with a file format of your choice), let's name it 'sphere_styleS.dat'

1.1.2 Loop over the different light-source positions. We use 48 positions, all in the first quadrant (X>0, Y>0, Z=-d..+d). The other three quadrants are created by mirroring on the fly when the user moves the light-source interactively around the molecule (i.e. the user can choose 192 light-source positions, which is enough to hide the fact that the graphics are precalculated).

1.1.2.1 Loop over the three different atom shifts along the X axis: The atom is now ray-traced three times, horizontally shifted by 0, -s and +s. The first zero shift is used normally, and the latter two shifts are used for stereoscopic 3D graphics, where the images for left and right eye need different sphere graphics, so that spheres appear plastic and not flat.

1.1.2.1.1 Run POVRay to trace the sphere with color yellow and size 256x256 pixels, given the current light-source position and horizontal camera shift. For 4K and Apple retina displays, the sphere size should be increased to 512x512 pixels, and the steps below adjusted.

1.1.2.1.2 Load the POVRay output file

1.1.2.1.3 Move the sphere to the left side (for stereo modes, we can't simply create a 256*256 output file due to the shift -s and +s, and use 640*256 instead)

1.1.2.1.4 Clip the sphere bitmap size to 256x256 pixels

1.1.2.1.5 Loop over the 256 pixel lines

1.1.2.1.5.1 Find the first non-empty pixel from the left and set all empty pixels on the left to its color.

1.1.2.1.5.2 Find the first non-empty pixel from the right and set all empty pixels on the right to its color. The last two steps make sure that the background color

is removed from the sphere bitmap and does not accidentally appear during rendering with OpenGL.

1.1.2.1.6 To save space, compress the sphere bitmap from 24-bit true-color to an 8-bit intensity (considering the red and blue color channels only, the green channel equals the red, since the sphere was ray-traced in yellow).

1.1.2.1.7 Compress the 8-bit intensity further with Zlib

1.1.2.1.8 Store the Zlib compressed 256x256 8-bit intensity bitmap in file 'sphere_styleS.dat'

2 On application startup: set the default parameters for the graphics engine, for example by loading them from an *.ini file that stores the user's preferences

2.1 ply_lightdir – The direction (unit-)vector from the origin to the light-source for all PoLYgons.

2.2 atv_size – ATom Visualization Size - The size of atoms when they touch (and are clipped by) the view plane, in percent of the maximum size supported by the graphics engine [0..1]. Changing this size is equivalent to moving the near clipping plane.

2.3 atv_texture – The texture applied to atoms (plain, granite, sky reflection..)

2.4 atv_ambience – The amount of darkness caused by blocked ambient light [0..1]

2.5 atv_shadow – The amount of darkness caused by shadows [0..1]

3 Create the application window

4 Initialize the graphics engine

4.1 Determine ply_eyedis, the distance between the eye (=camera) and the view plane in pixels, which depends on the size and aspect ratio of the application window: If WindowSize.x*0.75> WindowSize.y, then ply_eyedis=WindowSize.x, else ply_eyedis=WindowSize.y/0.75.

4.2 Determine the scaling factor from atom coordinates [in femtometers] to screen pixels: atv_fmtopix=8e-7*ply_eyedis*atv_size, e.g. if the window size is 1024*768 and atoms are shown with full size, then an atom sphere with radius 1.5 Å gets a radius of 123 pixels for visualization.

4.3 Allocate a 1024*1024 OpenGL texture atv_viewtex (Figure 1B), where the precalculated sphere images will be stored (or 2048*2048 for high-res screens). If stereoscopic 3D is enabled, allocate another texture to store sphere images as seen from the other eye (atv_viewtex2).

4.4 Create a table 'defradtab' of defined sphere radii (in pixels) that will be present in atv_viewtex (Figure 1B), we use [1,2,3,4,5,6,7,8,9,10,11,12,14,16,18,20,22,24,26,28,30,32,64,127], so the largest atom that can be displayed (without zooming the texture and reducing the resolution)

has a radius of 127 pixels.

4.5 Define ATV_COLORS, the number of standard colors available, we use ATV_COLORS=7: blue, magenta, red, yellow, green, cyan and grey (Fig.1B). Atoms with non-standard colors can be created by blending two standard colors using multi-texturing and a variable blending factor.

4.6 Loop over the sphere radii r in defradtab:

4.6.1 Calculate the alpha mask for a filled circle of radius r (which will later be filled with the 2D image of the atom sphere). This is just a bitmap of 8-bit values, 2r*2r pixels large, with values indicating the fraction of the pixel covered by the filled circle (ranging from 0 (pixel is totally outside circle) to 255 (pixel is totally inside circle)).

4.6.2 Loop over the ATV_COLORS, and for each find the next empty spot in texture atv_viewtex, copy the alpha mask to the texture's alpha channel at this spot (and also to atv_viewtex2 if present).

4.7 Load the Zlib compressed file with the 8-bit intensity bitmaps of size 256*256 'sphere_styleS.dat', where 'S' is the chosen atom texture atv_texture. This step is repeated whenever the user changes the atom texture.

4.8 Fill the textures atv_viewtex/atv_viewtex2 with actual sphere images. This step is repeated whenever the user chooses a new atom texture or changes the light-source position.

4.8.1 Loop over the one or two sphere images stored in 'sphere_styleS.dat' (1 normally, 2 in stereoscopic 3D mode):

4.8.1.1 Extract and uncompress the 8-bit intensity bitmap corresponding to the current light-source position using Zlib. Potentially mirror the bitmap horizontally and/or vertically (if the light-source is not in the first quadrant).

4.8.1.2 Loop over the sphere radii r in defradtab:

4.8.1.2.1 Shrink the 256*256 sphere image down to size 2r*2r

4.8.1.2.2 Loop over the ATV_COLORS standard colors i:

4.8.1.2.2.1 Convert the 8-bit intensity bitmap to an RGB color bitmap with color i.

4.8.1.2.2.2 Copy the bitmap to its position in atv_viewtex or atv_viewtex2, the alpha channel has been initialized previously.

4.8.2 The result should now look like in Figure 1B.

4.9 Precalculate Z-buffers for drawing intersecting atoms:

4.9.1 Create a 256*256 Z-buffer of 8-bit values 'atv_zbuffbak', which stores the depth values

of a sphere with radius 127, in practice the relative Z-coordinate of the sphere surface. 127 is assigned to the pixels at the very front (sphere center), 0 is assigned to the most distant pixels including those outside the sphere.

4.9.2 Loop over the chemical elements i:

4.9.2.1 Determine ElementRadius[i], the space filling visualization radius of this element in pixels (we use Van der Waals radius [in fm] * 0.7 * atv_fmtopix, which proves most useful for visualization, since hydrogens can still be seen outside their heavy atoms, and hydrogen-bonded hydrogens nicely touch the acceptor).

4.9.2.2 Has the same ElementRadius[i] already been used for a preceding chemical element? Then skip this element and reuse the Z-buffers of the preceding element. To save memory, elements with similar radii are grouped together for visualization purposes.

4.9.2.3 Loop over the radii r from 1 to ElementRadius[i]

4.9.2.3.1 Create a copy of the 256*256 Z-buffer atv_zbuffbak

4.9.2.3.2 Shrink it down to size 2r*2r

4.9.2.3.3 Scale the Z-values in the Z-buffer with ElementRadius[i]/127.

4.9.2.3.4 Store the Z-buffer for later use (when intersecting spheres are drawn)

4.10 Precalculate the atom densities for ambient lighting. A very low-res 8-bit 3D density grid (2.62 Å spacing) of the scene will be created to determine the amount of light reaching each atom from the six main directions (+X,-X,+Y,-Y,+Z,-Z). Densities to fill this 3D grid are precalculated for the three atom styles 'space filling', 'balls&sticks' and 'sticks', with radii of 1.2 Å, 0.95 Å and 0.85 Å, respectively (chosen empirically to give a convincing visual result). Since atoms are thus smaller than the grid spacing of 2.62 Å, the density needs to be added with 'sub-grid-cell' accuracy. We define this 'sub-grid-cell' accuracy with 2 bits, i.e. four slightly shifted densities along each axis are precalculated for each of the three atom styles, yielding 4*4*4*3 = 192 different atom densities (the atom density index 'ADIdx' can then be calculated from the atom coordinate and atom style using simple shifts and logical operations as described in detail later). The actual density values are obtained by calculating the fraction of the grid cell that is inside the atom and multiplying the result with 112. Since an atom with radius 1.2 Å and volume 7.24 Å^3 occupies at most 7.24/2.62^3 = 40% of a grid cell, the maximum density added is thus 112*0.4 = 45. So if more than 255/45 = ~5.7 atoms are present in a cell, the limit imposed by the 8-bit data-type is reached, the grid cell becomes completely light blocking. As described later, the densities will be integrated along the six main directions,

finally ambient light reaching each atom will be interpolated from the integrated grid.

4.11 Precalculate the atom circle masks for shadow calculation. A low-res 16-bit Z-buffer of the scene (with a resolution of 0.164*0.164 Å per pixel) will be drawn as seen from the direction of the light-source (Figure 1D). For each atom, one can then count the fraction of pixels with a Z-value smaller or equal than the atom's own Z-value, which is the fraction of light that reaches the atom. The shape of the atoms drawn in the Z-buffer is stored in three circle masks (16-bit bitmaps that contain 0 for pixels outside the circle and 0xffff for pixels inside the circle and are used as an AND mask when filling the Z-buffer (described later)). The sizes of the three circle masks are 15*15 pixels (used for space filling atoms), 11*11 pixels (for ball&sticks atoms), and 7*7 pixels (for atoms shown as sticks). So an 'X' corresponds to 0xffff, and a '.' to 0:

```
.....xxxxx.....   ....xxx....   ..xxx..
...xxxxxxxxx...   ..xxxxxxx..   .xxxxx.
..xxxxxxxxxxx..   .xxxxxxxxx.   xxxxxxx
.xxxxxxxxxxxxx.   .xxxxxxxxx.   xxxxxxx
.xxxxxxxxxxxxx.   xxxxxxxxxxx   xxxxxxx
xxxxxxxxxxxxxxx   xxxxxxxxxxx   .xxxxx.
xxxxxxxxxxxxxxx   xxxxxxxxxxx   ..xxx..
xxxxxxxxxxxxxxx   .xxxxxxxxx.
xxxxxxxxxxxxxxx   .xxxxxxxxx.
xxxxxxxxxxxxxxx   ..xxxxxxx..
.xxxxxxxxxxxxx.   ....xxx....
.xxxxxxxxxxxxx.
..xxxxxxxxxxx..
...xxxxxxxxx...
.....xxxxx.....
```

4.12 Initialize the 256 element table atv_darknessfogpos, which converts an 8-bit darkness value (from shadows and ambient lighting, see further below) to a 32-bit float (the Z-position provided as fog coordinate in a GL_FOG_COORDINATE_ARRAY, yielding the expected darkness considering the current fog equation).

5 Run the application's main loop

5.1 Evaluate keyboard and mouse input to let the user interact with the scene

5.2 For any newly added atoms i: Set the radius Atom[i].Radius [in pixels]. If Atom[i].Style is 'stick', choose a user-defined stick radius. Otherwise consider the chemical element: Atom[i].Radius = ElementRadius[Atom[i].Element]. If Atom[i].Style is 'ball&stick', scale the radius down by a user-defined factor.

5.3 Calculate ambient lighting:

5.3.1 Loop over all objects ('objects' are independent ranges of atoms that can be moved and

rotated separately, e.g. one object per PDB file loaded, or one object per NMR ensemble member). Have atoms in the object changed their relative positions since the last loop? Have atoms been added or deleted? Then ambient lighting has changed and needs to be updated for this object now:

5.3.1.1 Loop over the heavy atoms i in the object to determine the bounding box in units of [femtometers] from the atom coordinates in the local coordinate system of the original PDB file (Atom[i].LocalPos).

5.3.1.2 Convert the size of the bounding box from [fm] to [grid units], 1 grid unit is 1<<18 fm, i.e. 2.62 Å (see initialization).

5.3.1.3 Allocate a 3D grid of 8-bit unsigned bytes with the dimensions of the bounding box. This grid stores the densities.

5.3.1.4 Loop over the heavy atoms i in the object:

5.3.1.4.1 Determine the grid coordinates of the cube that contains the atom center: CubePos=(Atom[i].LocalPos-BoundingBoxStart)>>18.

5.3.1.4.2 Determine the relative atom position within the grid cube, which is in the range 0..3: AtomRelPos=((Atom[i].LocalPos-BoundingBoxStart)>>16)&3.

5.3.1.4.3 Determine ADIdx, the index into the atom density data for this atom. The index depends on the atom style (0=space filling, 1=ball&stick, 2=stick) and the relative atom position, and is in the range [0..191] (see initialization above).
ADIdx=AtomRelPos.x+AtomRelPos.y*4+AtomRelPos.z*16+Atom[i].Style*64

5.3.1.4.4 Use ADIdx to look up the step from CubePos to the front/left/bottom cube touched by the atom, the number of grid cubes along X/Y/Z affected by the atom, and the atom density data to be added to the ambience grid.

5.3.1.4.5 Add the atom density data, using SIMD vector instructions for adding unsigned bytes with saturation (e.g. paddusb).

5.3.1.5 Allocate a second 3D grid of unsigned bytes with the dimensions of the bounding box. This grid stores integrated densities.

5.3.1.6 Loop over the density grid four times: up the Y-axis, down the Y-axis, up the Z-axis, down the Z-axis

5.3.1.6.1 Proceed along the current axis direction, adding up the densities in an accumulation register (again using SIMD vector instructions for adding unsigned bytes with saturation), and continuously storing the accumulator register in the second grid, which thus contains integrated densities, i.e. the amount of light

blocked up to this point.

5.3.1.6.2 Loop over the heavy atoms i in the object:

5.3.1.6.2.1 Determine again the grid coordinates of the cube that contains the atom center: CubePos=(Atom[i].LocalPos-BoundingBoxStart)>>18.

5.3.1.6.2.2 Determine again the relative atom position within the grid cube, this time without discarding bits: AtomRelPos=(Atom[i].LocalPos-BoundingBoxStart)&((1<<18)-1).

5.3.1.6.2.3 Use AtomRelPos as an interpolation factor to interpolate the integrated density at the position of the atom from the surrounding eight grid points at CubePos.x/.y/.z, CubePos.x+1/.y/.z, CubePos.x/.y+1/.z etc.

5.3.1.6.2.4 Store the interpolated integrated density as one of six per-atom darkness values for later use

5.3.1.7 At this point, four darkness values (=interpolated integrated densities) per atom are available, two are still missing: up the X-axis and down the X-axis. These could not be obtained easily from the density grid, because integration along X would need to happen 'horizontally' within a SIMD register. Instead, the entire procedure above is simply repeated with a flipped grid:

5.3.1.8 Flip the X- and Y-axes of the bounding box

5.3.1.9 Allocate a 3D grid of unsigned bytes with the dimensions of the flipped bounding box. This grid stores densities.

5.3.1.10 Loop over the density grid two times: up the X-axis and down the X-axis

5.3.1.10.1 Obtain the remaining two interpolated integrated densities (=darkness values) as described above, flipping atom X- and Y-coordinates to match the flipped grid axes.

5.3.2 Now six darkness values 'DirectionDarkness[0..5]' are available for each atom, that define how much density lies in the path of the light along each of the six main directions.

5.3.3 Loop over all objects to determine the final ambient darkness value, which depends on the position of the eye (=camera) with respect to the object's six main direction, and must thus be recalculated if the object is rotated.

5.3.3.1 Determine EyeDirection, the direction vector from the object to the eye in the object's local coordinate system (where the darkness values were determined along the six main directions MainDirection[0..5], i.e. (1,0,0), (-1,0,0), (0,1,0) etc.

5.3.3.2 Calculate the scaling factors i for the six darkness values:

Scale[i]=(MainDirection[i]*EyeDirection+1)*0.5

5.3.3.3 Loop over the atoms i in the object:

5.3.3.3.1 Calculate the final ambient darkness value: AmbientDarkness[i] = DirectionDarkness[i][0]*Scale[0]+ DirectionDarkness[i][1]*Scale[1]+..... +DirectionDarkness[i][5]*Scale[5]

5.4 Calculate shadows:

5.4.1 Get a transformation matrix 'LightTransMatrix' that rotates ply_lightdir onto the negative Z-axis, so that the light shines from behind, this is the coordinate system of the light-source.

5.4.2 Loop over the objects i:

5.4.2.1 Transform the global position of object i, Obj[i].GlobalPos (in [fm]), with LightTransMatrix, yielding a new position Obj[i].Pos in the coordinate system of the light-source.

5.4.2.2 Have atom coordinates in the object changed since last time? If yes, re-determine Obj[i].Radius (in [fm]), the radius of the sphere that is centered at the position of the object and encloses all atoms in the object.

5.4.3 Use the radii of the enclosing spheres to cluster the objects, forming groups of those that cast shadows onto each other.

5.4.4 Loop over the groups of objects:

5.4.4.1 Sort the objects i in the group based on the smallest Z-coordinate covered (Obj[i].Pos.z-Obj[i].Radius), lowest comes first.

5.4.4.2 For each object i in the group, create a transformation matrix Obj[i].TransMatrix from the local (PDB) coordinate system used by the atoms in the object (Atom[].LocalPos) to the coordinate system of the light-source. This transformation includes an initial rotation, a translation to Obj[i].GlobalPos and finally LightTransMatrix.

5.4.4.3 Shift the objects i in the group along Z (and adjust Obj[i].TransMatrix accordingly) so that the first object starts at Z=0 (Obj[first].Pos-z-Obj[first].Radius=0) and that there are no gaps between the enclosing spheres along Z (we soon need to create a common Z-buffer for all objects in the group, and large gaps between objects would waste Z-buffer resolution).

5.4.4.4 Loop over the objects i in the group to determine the Z-range covered: ZRange=max(ZRange,Obj[i].Pos.z+Obj[i].Radius).

5.4.4.5 Adapt each Obj[i].TransMatrix to scale the Z-coordinate with -32767/ZRange and add 32767. If the local atom coordinates Atom[i].LocalPos in an object are multiplied with this matrix, the resulting Z-coordinates will fall into the range [0..32767] and fit into a 16 bit signed Z-buffer. Note the inversion, the closest atom gets the highest Z-coordinate.

5.4.4.6 Loop over the objects in the group to determine the bounding rectangle along X and Y in units of [fm], e.g. BoundingRectStart.x = min(BoundingRectStart.x,Obj[i].Pos.x-Obj[i].Radius) etc.

5.4.4.7 Convert the size of the bounding rectangle from [fm] to [Z-buffer units], 1 Z-buffer unit is 1<<14 fm, i.e. ~0.164 Å (see initialization).

5.4.4.8 Allocate 2D 16bit Z-buffers of signed words with the dimensions of the bounding rectangle, one for each thread that will work on shadows. Using more than four threads is not beneficial.

5.4.4.9 Distribute the atoms in the objects of the current cluster among the threads.

5.4.4.10 Spawn threads to fill the Z-buffers, and in each thread...

5.4.4.10.1 Clear the 2D Z-buffer belonging to this thread with zeroes (i.e. the most distant value after the sign flip above).

5.4.4.10.2 Loop over the heavy atoms i belonging to this thread:

5.4.4.10.2.1 Determine which object j the atom i belongs to.

5.4.4.10.2.2 Transform atom i to the coordinate system of the light: Atom[i].LightPos = Obj[j].TransMatrix * Atom[i].LocalPos.

5.4.4.10.2.3 Determine the Z-buffer X/Y coordinates of the atom: ZBufferPos=(Atom[i].LightPos-BoundingRectStart)>>14.

5.4.4.10.2.4 Look at the atom style to determine the diameter of the filled circle that will be drawn into the Z-buffer (the circle mask). We use 15 pixels for space filling balls, 11 pixels for balls&sticks, and 7 pixels for sticks (see setup).

5.4.4.10.2.5 Update the Z-buffer X/Y coordinates by subtracting the radius of the filled circle.

5.4.4.10.2.6 Loop over the 7x7, 11x11 or 15x15 square in the Z-buffer that encloses the current atom using indices k,l:

5.4.4.10.2.6.1 Update the Z-buffer: ZBuffer[X+k][Y+l]= max(ZBuffer[X+k][Y+l], Atom[i].LightPos.z & AtomCircleMask[k][l]). AtomCircleMask

contains the precalculated filled circle (see setup), each pixel is either 0 or 0xffff, and used as an AND-mask, yielding either 0 (for pixels outside the circle) or Atom[i].LightPos.z (for pixels inside the circle). The corresponding SIMD vector instructions are pmaxsw and pand.

5.4.4.11 Combine the Z-buffers of the threads by keeping the maximum values, the result should look as shown in Figure 1D, with Z-buffer value 0 mapped to white, and 32767 mapped to black.

5.4.4.12 Spawn threads to determine the amount of light reaching each atom, and in each thread...

5.4.4.12.1 Loop over the atoms i belonging to this thread:

5.4.4.12.1.1 Determine the Z-buffer X/Y coordinates of the atom: ZbufferPos=(Atom[i].LightPos-BoundingRectStart)>>14.

5.4.4.12.1.2 Count all Z-buffer pixels within the large atom circle (15 pixels diameter) that are smaller or equal Atom[i].LightPos.z, then divide by the number of pixels in the circle to obtain the fraction of the atom that is lighted. Note that the large circle is also used for smaller atoms (shown as sticks or balls & sticks), because the larger area reduces flicker.

5.4.4.12.1.3 Calculate the ShadowDarkness[i] = 1 - lighted fraction.

5.5 Loop over the atoms i to calculate the final darkness: Atom[i].Darkness= max(AmbientDarkness[i]* atv_ambience, ShadowDarkness[i]*atv_shadow)

5.6 Loop over the objects and create new transformation matrices Obj[i].TransMatrix from the local (PDB) coordinate system used by the atoms in the object (Atom[].LocalPos in [fm]) to the global coordinate system in [pixels]. This transformation includes an initial rotation, a translation to Obj[i].GlobalPos, and a conversion from fm to pixels (atv_fmtopix).

5.7 Loop over the atoms i to transform their coordinates:

5.7.1 Transform the atom from the local coordinate system within its object j to the common global coordinate system: Atom[i].GlobalPos [in pixels] = Obj[j].TransMatrix * Atom[i].LocalPos [in fm]

5.7.2 Project the global atom coordinates onto the view plane (the 'perspective division'): Atom[i].ProjectedPos.x=Atom[i].GlobalPos.x*ply_eyedis/ (Atom[i].GlobalPos.z+ply_eyedis), and Atom[i].ProjectedPos.y=-Atom[i].GlobalPos.y*ply_eyedis/ (Atom[i].GlobalPos.z+ply_eyedis). Note that the Y-coordinate is reversed, because

projected screen coordinates increase from top to bottom.

5.7.3 Determine the projected atom radius[pixels]: Atom[i].ProjectedRadius=Atom[i].Radius* ply_eyedis/(Atom[i].GlobalPos.z+ply_eyedis) .

5.7.4 Loop over the four screen sides (left, right, top, bottom) and check if the atom is entirely off-screen on this side (considering Atom[i].ProjectedPos and Atom[i].ProjectedRadius). If yes, set the corresponding Cohen Sutherland clipping bit to 1.

5.7.5 If abs(Atom[i].GlobalPos.z)<Atom[i].Radius, then add the atom to atv_vpcliptab, the table of atoms clipped by the view plane.

5.8 Draw the sticks between atoms shown as sticks or balls&sticks:

5.8.1 Enable texture mapping

```
BindTexture(GL_TEXTURE_2D,atv_viewtex);
glTexEnvi(GL_TEXTURE_ENV,GL_TEXTURE_ENV_MODE,GL_REPLACE);
glEnable(GL_TEXTURE_2D);
glEnable(GL_ALPHA_TEST);
```

5.8.2 Enable vertex arrays:

```
glEnableClientState(GL_TEXTURE_COORD_ARRAY);
glEnableClientState(GL_COLOR_ARRAY);
```
(for blending factors)

5.8.3 Initialize the second texture unit (unit 1). We need multi-texturing to create colors other than the ATV_COLORS standard colors stored in atv_viewtex. We simply blend two of the standard colors together, using the color from GL_COLOR_ARRAY as blending factor.

```
glActiveTexture(GL_TEXTURE1);
glBindTexture(GL_TEXTURE_2D,atv_viewtex);
```

5.8.3.1 In texture unit 1, we GL_COMBINE by GL_INTERPOLATING the previous RGB/alpha values from texture unit 0...

```
glTexEnvi(GL_TEXTURE_ENV,GL_TEXTURE_ENV_MODE,GL_COMBINE)
glTexEnvi(GL_TEXTURE_ENV,GL_COMBINE_RGB,GL_INTERPOLATE);
glTexEnvi(GL_TEXTURE_ENV,GL_COMBINE_ALPHA,GL_INTERPOLATE)
glTexEnvi(GL_TEXTURE_ENV,GL_SRC0_RGB,GL_PREVIOUS);
glTexEnvi(GL_TEXTURE_ENV,GL_SRC0_ALPHA,GL_PREVIOUS);
glTexEnvi(GL_TEXTURE_ENV,GL_OPERAND0_RGB,GL_SRC_COLOR);
glTexEnvi(GL_TEXTURE_ENV,GL_OPERAND0_ALPHA,GL_SRC_ALPHA)
```

5.8.3.2 ..with those from the current texture unit 1...

```
glTexEnvi(GL_TEXTURE_ENV,GL_SRC1_RGB,GL_TEXTURE);
glTexEnvi(GL_TEXTURE_ENV,GL_SRC1_ALPHA,GL_TEXTURE);
glTexEnvi(GL_TEXTURE_ENV,GL_OPERAND1_RGB,GL_SRC_COLOR);
glTexEnvi(GL_TEXTURE_ENV,GL_OPERAND1_ALPHA,GL_SRC_ALPHA)
```

5.8.3.3 ...using the GL_PRIMARY_COLOR as interpolation factor:

```
glTexEnvi(GL_TEXTURE_ENV,GL_SRC2_RGB,GL_PRIMARY_COLOR);
glTexEnvi(GL_TEXTURE_ENV,GL_SRC2_ALPHA,GL_PRIMARY_COLOR)
glTexEnvi(GL_TEXTURE_ENV,GL_OPERAND2_RGB,GL_SRC_COLOR);
glTexEnvi(GL_TEXTURE_ENV,GL_OPERAND2_ALPHA,GL_SRC_ALPHA)
glEnableClientState(GL_TEXTURE_COORD_ARRAY);
```

5.8.4 Is the extension GL_EXT_fog_coord present? We need it to set the darkness caused by ambient lighting, shadows and fog. Especially OpenGL/ES is lacking it. So if it is missing, use texture unit 2 to emulate it using a 1x256 decal texture 'ply_fogtex', which is used to blend with the fog color:

```
glActiveTexture(GL_TEXTURE2);
glBindTexture(GL_TEXTURE_2D,ply_fogtex);
glEnable(GL_TEXTURE_2D);
glTexEnvi(GL_TEXTURE_ENV,GL_TEXTURE_ENV_MODE,GL_DECAL);
glClientActiveTexture(GL_TEXTURE2);
glEnableClientState(GL_TEXTURE_COORD_ARRAY);
```

5.8.5 Switch back to texture unit 0:

```
glActiveTexture(GL_TEXTURE0);
glClientActiveTexture(GL_TEXTURE0);
```

5.8.6 Is the extension GL_EXT_fog_coord present? Then activate it...

```
glFogi(GL_FOG_COORDINATE_SOURCE,GL_FOG_COORDINATE);
glEnableClientState(GL_FOG_COORDINATE_ARRAY);
```

5.8.7 Draw only pixels with Alpha>0.5:

```
glAlphaFunc(GL_GREATER,0.5);
```

5.8.8 Enable the Z-buffer

```
glDepthFunc(GL_LEQUAL);
glEnable(GL_DEPTH_TEST);
```

5.8.9 Create a queue where multiple threads can add draw buffers with geometry that is ready to be sent to OpenGL

5.8.10 Spawn N threads to draw the sticks, and in..

5.8.10.1 Thread 0, the consumer: Check the queue of draw buffers, and submit any waiting buffers (consisting of a vertex buffer and an index buffer) to OpenGL using primitive GL_TRIANGLE_STRIP.

5.8.10.2 Thread 1..N-1, the workers: Loop over the atoms i assigned to this thread:

5.8.10.2.1 Is atom i in front of the view plane and not hidden by the user? Then loop over its covalently bound atoms j:

5.8.10.2.1.1 Is one of the two binding partners styled as stick or ball&stick? And is the bound atom not hidden by the user? And has the bound atom j a larger Z? (Or the same Z and a higher atom number?) And is the bitwise AND of the two binding partner's Cohen Sutherland clipping bits zero? Then draw the stick:

5.8.10.2.1.1.1 Get the normalized direction vector from atom i to atom j: Vec1 = Normalized(Atom[j].GlobalPos-Atom[i].GlobalPos).

5.8.10.2.1.1.2 Get the 2D normal vector of the projected direction vector from atom i to atom j: Vec2 = Normalized(Atom[j].ProjectedPos.y-Atom[i].ProjectedPos.y, Atom[i].ProjectedPos.x - Atom[j].ProjectedPos.x)

5.8.10.2.1.1.3 The cylinder (=stick) vertices lie in planes whose normal vector is Vec1. Get two vectors spanning this plane: the first is Vec2, and the second is Vec3 = Normalized(CrossProduct(Vec2,Vec1)).

5.8.10.2.1.1.4 Determine the level of detail (LOD), i.e. how many quads (=rectangles consisting of 2 triangles) will be used to approximate the bond cylinder. If Atom[i].ProjectedRadius>9, we draw 5 quads with 12 vertices (i.e. 6 steps*2 sides=12 vertices, 36 degrees apart, since only the front side of each cylinder is drawn). If Atom[i].ProjectedRadius>3, we use 3 quads (4 steps*2=8 vertices, 60 degree apart), and one single quad otherwise.

5.8.10.2.1.1.5 Determine the distance between the start of a stick cylinder attached to atom i and atom i (1 is subtracted to move a bit closer): StickDistance_i = max(0,sqrt(sqr(Atom[i].Radius)-sqr(StickRadius))-1).

5.8.10.2.1.1.6 Determine the distance between the start of a stick cylinder attached to atom j and atom j:

StickDistance_j = max(0,sqrt(sqr(Atom[j].Radius)-sqr(StickRadius))-1).

5.8.10.2.1.1.7 Loop over the steps, k=0..steps-1, calculate the vertex positions:

5.8.10.2.1.1.7.1 First the angle along the cylinder side surface:

Alpha = k*(Pi/(steps-1))

5.8.10.2.1.1.7.2 Then the position on the cylinder side surface: Pos=Vec2*cos(Alpha)*StickRadius+Vec3*sin(Alpha)*StickRadius, use a lookup table to speed this up.

5.8.10.2.1.1.7.3 Calculate the corresponding texture coordinate: TexPos.x(.y)= Pos.x(.y) * 0.97 * SphereImageRadius / StickRadius + SphereImageRadius, where SphereImageRadius is the radius of the ATV_COLORS largest sphere images in atv_viewtex. The empirical factor 0.97 makes sure that the texture position is not too close to the border of the sphere image.

5.8.10.2.1.1.7.4 Calculate the corresponding vertex positions at atoms i and j: Pos_i=Pos+Atom[i].GlobalPos+StickDistance_i*Vec1, Pos_j=Pos+Atom[j].GlobalPos-StickDistance_j*Vec1.

5.8.10.2.1.1.7.5 Store the vertex at atom i in the vertex buffer: Vertex.Pos = Pos_i, Vertex.TexPos1 = TexPos+ColorOffset1i, Vertex.TexPos2 = TexPos+ColorOffset2i, Vertex.Color=BlendingFactor_i (the color of atom i is created using multi-texturing, by blending two sphere images in texture atv_viewtex starting at ColorOffset1i and ColorOffset2i with BlendingFactor_i. The last three values are read from a look up table, using Atom[i].Color as index). Vertex.FogPos = atv_darknessfogpos[Atom[i].Darkness].

5.8.10.2.1.1.7.6 If atoms i and j have different colors, store two vertices halfway between, with position (Pos_i+Pos_j)*0.5. Except for the position, the first vertex is the same as the one above, and the second vertex is the same as the one below.

5.8.10.2.1.1.7.7 Store the vertex at atom j in the vertex buffer: Vertex.Pos = Pos_j, Vertex.TexPos1 = TexPos+ColorOffset1j, Vertex.TexPos2 =

TexPos+ColorOffset2j, Vertex.Color=BlendingFactor_j (as above). Vertex.FogPos = atv_darknessfogpos[Atom[j].Darkness]

5.8.10.2.1.1.8 Finish the stick by adding indices to the index buffer. If the index buffer is not empty, first add degenerate triangle indices to jump from the previous stick to the current one.

5.8.10.2.2 If the vertex buffer is full or the last atom has been reached, add it to the queue (to be drawn by thread 0) and fill the next buffer or stop.

5.8.11 Disable texture mapping, vertex arrays, alpha blending, depth test, restore depth func.

5.9 If antialiasing is enabled (and atoms will be drawn with alpha-blending enabled to get perfectly smooth spheres, independent of any full-scene antialiasing provided by the GPU): spawn multiple threads to sort the atoms by their Atom[i].GlobalPos.z, e.g. by dividing the Z-range into multiple small intervals, and using a simple insertion sort within each interval.

5.10 Draw the atom spheres:

5.10.1 Repeat the steps done for drawing sticks above: Enable texture mapping and vertex arrays, initialize the second texture unit, deal with GL_EXT_fog_coord. There is mainly one difference: If antialiasing is enabled, and atoms have been depth-sorted, don't draw only pixels with Alpha>0.5 (see above), but do real alpha blending instead:

```
glEnable(GL_BLEND);
glBlendFunc(GL_SRC_ALPHA,GL_ONE_MINUS_SRC_ALPHA);
glAlphaFunc(GL_GREATER,0.0);
```

5.10.2 Create a queue where multiple threads can add draw buffers.

5.10.3 Spawn N threads to draw the spheres. If antialiasing is enabled, we need to draw the depth-sorted spheres in order, so N is at most 2, more threads would not help. And in..

5.10.3.1 Thread 0, the consumer: Check the queue of draw buffers, and submit any waiting buffers (consisting of a vertex buffer and an index buffer) to OpenGL using primitive GL_TRIANGLE_STRIP.

5.10.3.2 Thread 1..N-1, the workers: If antialiasing is enabled and atoms have been depth-sorted before, loop over the atoms i in the sort table, otherwise loop over the atoms i assigned to this thread:

5.10.3.2.1 Is atom i in front of the view plane and not hidden by the user and not off screen (Cohen Sutherland clipping bits are zero)? Then draw it:

5.10.3.2.1.1 Look up the texture coordinates of the smallest two sphere images in atv_viewtex that are larger than Atom[i].ProjectedRadius (mip-mapping) and

need to be blended to produce the color of the current atom. Each of the two sphere images has actually two texture coordinates: the top left corner 'tl' and the bottom right corner 'br', so we have four texture coordinates in total: ColorOffset1tl, ColorOffset1br, ColorOffset2tl and ColorOffset2br.

5.10.3.2.1.2 Is the atom styled as stick or ball&stick? Or does the atom have no covalent bonds? Then we do not consider intersections with other atoms and can...

5.10.3.2.1.2.1 Draw a non-intersecting atom, i.e. a single quad (two triangles) by storing the four corner vertices in the vertex buffer (Figure 1C, oxygen and hydrogen 1):

5.10.3.2.1.2.1.1 Store top left corner: Vertex.Pos = (Atom[i].GlobalPos.x-Atom[i].Radius, Atom[i].GlobalPos.y+Atom[i].Radius, Atom[i].GlobalPos.z),Vertex.TexPos1=ColorOffset1tl, Vertex.TexPos2=ColorOffset2tl,Vertex.Color=BlendingFactor_i, Vertex.FogPos=atv_darknessfogpos[Atom[i].Darkness] (these vertex components are described in detail in the section about stick drawing).

5.10.3.2.1.2.1.2 Top right corner: Vertex.Pos = (Atom[i].GlobalPos.x+ Atom[i].Radius, Atom[i].GlobalPos.y+Atom[i].Radius, Atom[i].GlobalPos.z), Vertex.TexPos1=(ColorOffset1br.x,ColorOffset1tl.y), Vertex.TexPos2=(ColorOffset2br.x,ColorOffset2tl.y), rest is the same.

5.10.3.2.1.2.1.3 Bottom left corner: Vertex.Pos=(Atom[i].GlobalPos.x-Atom[i].Radius,Atom[i].GlobalPos.y-Atom[i].Radius, Atom[i].GlobalPos.z), Vertex.TexPos1=(ColorOffset1tl.x,ColorOffset1br.y), Vertex.TexPos2=(ColorOffset2tl.x,ColorOffset2br.y).

5.10.3.2.1.2.1.4 Bottom right corner: Vertex.Pos=(Atom[i].GlobalPos.x+ Atom[i].Radius,Atom[i].GlobalPos.y-Atom[i].Radius, Atom[i].GlobalPos.z),Vertex.TexPos1=ColorOffset1br, Vertex.TexPos2=ColorOffset2br, rest is again the same.

5.10.3.2.1.3 Otherwise (if the atom is shown in space filling style), it can potentially intersect with other atoms and we need to...

5.10.3.2.1.3.1 Draw a potentially intersecting atom (Figure 1C, hydrogen 2):

5.10.3.2.1.3.1.1 Calculate the screen coordinates of the top left corner and store it in 2D vectors StartLeftEdge=LastLeftEdge= Atom[i].ProjectedPos-Atom[i].ProjectedRadius.

5.10.3.2.1.3.1.2 Calculate the screen coordinates of the top right corner, and store it in 2D vectors StartRightEdge=LastRightEdge= (LeftEdge.x+Atom[i].ProjectedRadius*2,LeftEdge.y)

5.10.3.2.1.3.1.3 Collect all the more distant atoms j (Atom[j].GlobalPos.z>Atom[i].GlobalPos.z or (Atom[j].GlobalPos.z ==Atom[i].GlobalPos.z and j>i)) that can influence the shape of atom i by intersection, i.e. those atoms that are closer along Z than their own radius (Atom[j].GlobalPos.z-Atom[i].GlobalPos.z< Atom[j].Radius) and whose sphere image touches atom i: Length(Atom[j].ProjectedPos-Atom[i].ProjectedPos)< Atom[i].ProjectedRadius+Atom[j].ProjectedRadius. The atoms j could be found quickly with a neighbor search grid, but it turns out that the trivial approach to just look at covalently bound atoms is good enough. If no atom j is found, draw a non-intersecting atom instead.

5.10.3.2.1.3.1.4 Get pointers to the pre-calculated Z-buffers of atom i and the atoms j (there is one Z-buffer with 8-bit depth values for every Atom.ProjectedRadius, see initialization).

5.10.3.2.1.3.1.5 Loop over the Atom[i].ProjectedRadius*2 pixel lines of atom i:

5.10.3.2.1.3.1.5.1 Calculate the screen coordinates of the current left polygon edge, assuming no intersections: LeftEdge= (Atom[i].ProjectedPos.x-Atom[i].ProjectedRadius, LastLeftEdge.y+1)

5.10.3.2.1.3.1.5.2 Calculate the screen coordinates of the current right polygon edge, assuming no intersections: RightEdge= (Atom[i].ProjectedPos.x+Atom[i].ProjectedRadius, LastRightEdge.y+1)

5.10.3.2.1.3.1.5.3 Look up the positions of the left-most and right-most pixels of the current pixel line of atom i (tracing the sphere outline).

5.10.3.2.1.3.1.5.4 Loop over the probably intersecting atoms j:

5.10.3.2.1.3.1.5.4.1 Look up the positions of the left-most and right-most pixels of the corresponding pixel line of atom j. If they overlap with those of atom i, determine the corresponding pixel line in the Z-buffer of atom j, and compare the Z-buffer values (considering the Z-difference Atom[j].GlobalPos.z-Atom[i].GlobalPos.z) until the intersection is found. Update LeftEdge.x and RightEdge.x accordingly.

5.10.3.2.1.3.1.5.5 Check if LeftEdge lies roughly (+-1 pixel) on a line through StartLeftEdge and LastLeftEdge, and if RightEdge lies roughly on a line trough StartRightEdge and LastRightEdge. If this is not true, then the intersection curve has changed direction so much, that it can no longer be approximated by a straight line. We therefore need to emit four vertices to draw a part of the sphere (Figure 1C, hydrogen 2), as described above for drawing a non-intersecting atom. The only difference are Vertex.Pos, Vertex.TexPos1 and Vertex.TexPos2, which are no longer at the four corners of the atom, but at StartLeftEdge, StartRightEdge, LastLeftEdge, and LastRightEdge (these screen coordinates need to be transformed back to global coordinates and to texture coordinates). Finally set StartLeftEdge=LastLeftEdge and StartRightEdge=LastRightEdge.

5.10.3.2.1.3.1.5.6 Set LastLeftEdge=LeftEdge, and LastRightEdge= RightEdge.

5.10.3.2.2 If the vertex buffer is full or the last atom has been drawn, add it to the queue (to be drawn by thread 0) and fill the next buffer or stop.

5.10.4 Disable texture mapping, vertex arrays, alpha blending, depth test, restore depth func.

5.11 Handle clipped atoms: Loop over the atoms i that are cut by the view plane (stored in atv_vpcliptab), determine the radius of the clipping circle (CircleRadius = sqrt (sqr(Atom[i].Radius)-sqr(Atom[i].GlobalPos.z))) and draw it, potentially with a text indicating the chemical element, which thus becomes visible when the atom is cut open.

5.12 If stereoscopic 3D is active, repeat the drawing procedure using the second texture atv_viewtex2 and horizonally shifted atom coordinates.
